# Lymphocyte opioid receptors as innovative biomarkers of osteoarthritic pain, for the assessment and risk management of opioid tailored therapy, before hip surgery, to prevent chronic pain and opioid tolerance/addiction development: OpMarkArt (Opioids-Markers-Arthroprosthesis) study protocol for a randomized controlled trial

**DOI:** 10.1186/s13063-017-2363-z

**Published:** 2017-12-19

**Authors:** Valentina Malafoglia, Monica Celi, Carolina Muscoli, Sara Ilari, Filomena Lauro, Luigino Antonio Giancotti, Chiara Morabito, Maurizio Feola, Umberto Tarantino, William Raffaeli

**Affiliations:** 1 Institute for Research on Pain, ISAL Foundation, Torre Pedrera, RN Italy; 2 Policlinico Foundation Tor Vergata, University of Tor Vergata, Rome, Italy; 3 Institute of Research for Food Safety & Health (IRC_FSH), Department of Health Sciences, University ‘Magna Graecia’ of Catanzaro, Catanzaro, Italy; 4 San Raffaele Roma S.r.l., Roccelletta di Borgia, Catanzaro, Italy; 5 Department of Experimental Medicine, ‘Sapienza’ University of Rome, Rome, Italy

**Keywords:** Opioids, Opioid receptors, Lymphocytes, Arthroprosthesis, Hip surgery, Chronic pain, Biological markers, Tailored therapy, Tolerance, Addiction

## Abstract

**Background:**

The incidence of post-surgical chronic pain ranges between 20% and 40% in Europe. Osteoarthritis pain after prosthesis implantation is one of the most severe secondary syndromes, depending not only on surgery but also on organic changes before and after joints replacement. No data are available about risk factors. An excessive inflammatory response plays a central role but a best therapy is not defined yet. It is not clear whether opioid administration could influence post-surgical pain and lead to tolerance or addiction. Interestingly, the immune system, together with the nervous and peptidergic ones, is involved in hypersensibility. The connection across the three biological systems lies in the presence of opioid receptors on immune cells surface. Here, we show a method to analyze whether opioids could modulate lymphocytes, by proposing opioid receptors as biological markers to prevent chronic pain and opioid tolerance or addiction after hip surgery.

**Methods/design:**

After institutional independent ethics committee approval, 60 patients, in pain and undergoing hip surgery, will be enrolled in a single-blind, randomized, phase IV, pilot study. Pain treatment will be selected inside a class of non-steroidal anti-inflammatory drugs (NAISDs) or paracetamol or a class of opioids, into three medication arms: 25 mg tapentadol twice daily; 75 mg tapentadol twice daily; NSAIDs or paracetamol in accordance with surgeon’s custom. For each group, we will collect blood samples before, during and after surgery, to apply molecular analysis. We will perform lymphocyte opioid receptors genes and proteins expression and functional analysis. Data will be statistically analyzed.

**Discussion:**

This project has the potential to obtain a personalized diagnostic kit, by considering lymphocyte opioid receptors as biological markers. Starting from a simple blood sample, it will be possible to decide the best therapy for a single patient. Using a noninvasive approach, we expect to fix a daily standard dose and timing, before and after surgery, to bypass hip chronic pain and the insurgence of tolerance or addiction. The analysis of opioid receptors sensitivity will help to identify the best drug administration in each specific case (tailored therapy).

**Trial registration:**

ISRCTN, ISRCTN12559751. Retrospectively registered on 23 May 2017.

**Electronic supplementary material:**

The online version of this article (doi:10.1186/s13063-017-2363-z) contains supplementary material, which is available to authorized users.

## Background

Postoperative morbidity and persistent post-surgical pain is a complex unsolved problem, influencing patients’ outcomes and lives [1, 2]. It has been called a silent epidemic of great social impact, by involving patients for more than 4 months after surgery [3]. In particular, chronic pain after hip operation has very high direct costs because of the utilization of painkillers for years and a prolonged rehabilitation programme, to ensure the maintenance of patients’ motility with a sufficient quality of life. Always more frequently, there are also many indirect costs, owing to medical and legal disputes and disability allowances [4, 5]. Although the literature lacks sufficient information about risk factors and prevention or diagnostic molecular markers [6] and a real cause is still unknown, several elements, such as the type of pain, could influence the establishment of a permanent state of the disease [7]. Considering therapy, opioids are the most used drugs in this contest, even though there are not standard therapeutic guidelines and little is known about the right daily dosage or the exact timing of treatment. We also lack information about the influence of opioids on pre- and post-surgical pain. Thus, the debate around opioid utilization is always open, above all for the insurgence of side effects, as well as addiction and tolerance [8]. Interestingly, the immune system is strictly linked to the nervous and peptidergic systems because of the presence of opioid receptors on the surfaces of blood cells [9], although data are not available in relation to the insurgence of chronic pain. Because of this, since the 1990s, our group (ISAL Foundation) has been trying to study the opioid–lymphocyte interaction and its immune-hormonal impact [10, 11], by focusing on clinical and biochemical synergy across opioids, immune-hormonal system and pain pathologies [12–15]. Now, our aim is to analyze whether the utilization of opioids, whose use is largely validated in the literature and consolidated in the treatment of osteoarthritic pain, can have effects on lymphocytes and can modulate the onset of postoperative chronic pain. In particular, we want to propose opioid receptors as innovative biological markers, in order to analyze chronic pain predisposition or evolution and addiction or tolerance insurgence. Here we show a study protocol to set up the best pre-surgery opioid dosage for an individual patient, through biological analysis of opioid receptors.

## Methods

### Design

This is an interventional, pilot, single-blind, randomized, phase IV study. The protocol has been designed and financed by ISAL Foundation (Torre Pedrera, Rimini, Italy). Patients will be enrolled at Policlinico Tor Vergata, Rome, Italy. The clinical study has been approved by the institutional independent ethics committee of Policlinico Tor Vergata, in Rome, on 23 May 2016, under the name OpMarkArt (Opioids-Markers-Arthroprosthesis), experimental register 110/16. The trial has been retrospectively registered on the ISRCTN registry (ISRCTN12559751), on 23 May 2017.

### Patients

The orthopaedic specialist will enrol only patients who previously entered a waiting list for total hip replacement surgery, by following the scientific society guidelines for patient selection. The clinician will describe the research, by discussing any details about drugs and showing the goals of the study. Inclusion criteria allow the eligibility of male and female patients, who will undergo total hip arthroplasty for osteoarthritis or aseptic necrosis of the femoral head, are older than 18 and are selected in accordance with the orthopaedic specialist’s indications. During the baseline visit, patients who chronically take painkillers before inclusion will be excluded from the protocol, as well as patients with anamnestic adverse effect to NSAIDs or paracetamol or with gastric ulcers. Exclusion criteria also involve patients with unstable neurological pathologies, uncompensated diabetes, previous abdominal surgery with dynamic ileum risk, or viral infective pathologies, patients unable to fill the informed form or patients who need post-surgical mechanical ventilation or are waiting for secondary surgery. Withdrawal criteria allow consent to be removed at any time and for any reason. Patients who withdraw from the study will not be readmitted in the study. Before starting the study and during follow-up, physical examination will be performed, and the Harris Hip Score (Additional file 1) will be used to evaluate hip movement, stability, strength, the presence of any deformities, and joint and functional limitations. Radiographs of the axial pelvis and in anteroposterior will be obtained, to determine the extent of the degree of arthritis or necrosis; pain assessment will be conducted using the model inside the Harris Hip Score, by adding evaluations of pain intensity (numeric pain rating scale, Additional file 2) during orthostatic and clinostat posture. All eligible and consenting patients will sign a specific informed-consent form. Each allocated patient, after inclusion and exclusion criteria analysis and clinical evaluation, and on obtaining consent, will be randomly included in one of the three groups of medication, by a computational approach. Only the orthopaedist can access the names of the patients, who will be registered with a numeric sequential code, in order to protect confidentiality. All the information will be collected in an electronic file. The research biologists will be blind for patients’ personal information and therapy. They will receive patients’ samples and will record data by following the numeric sequential code. The orthopaedic team and the patients will know about the medications they are following.

### Procedure: clinical study

Patients will be divided into three groups of medication, administered in common practice: 25 mg tapentadol twice daily; 75 mg tapentadol twice daily; and NSAIDs or paracetamol in accordance with the surgeon’s customary practice. The inclusion of patients in the groups will be random and will be performed through computational approach. The research biologists will be blind to the assigned treatment. Patients included in the NSAIDs and paracetamol group will receive drugs according to common practice, considering the necessary dosage. Usually, they take NSAIDs as the first drug for a week then 1 g paracetamol twice daily plus 600 mg ibuprofen, as a rescue dose, if they feel pain. Placebo is not used. All patients with anamnestic adverse effects to NSAIDs or paracetamol or with gastric ulcer will not be enrolled. All the arms will follow the specific pharmacological plan for 30 days before surgery and 15 days after (if the patient feels pain). Each patient will be subjected to five blood sample collections at precise time points: the day of enrolment and starting therapy (T0); the 30th day of therapy, right before the moment of surgery (T2); the day after surgery (T3); 30 days after surgery (T4); and 60 days after surgery, in correspondence with the final assessment and follow-up (T5). T1 coincides with clinical examination, 15 days after the enrolment, without blood collection. On the day of surgery, patients will be monitored using standard practices, such as electrocardiography, oxygen saturation sensing, invasive and noninvasive blood pressure monitoring. Patients will receive a regional anaesthesia, by epidural catheter, during and after surgery. They will not receive any opiod drugs. During surgery, a bone marrow sample will be collected.

### Procedure: laboratory analysis

**Fig. 1:**
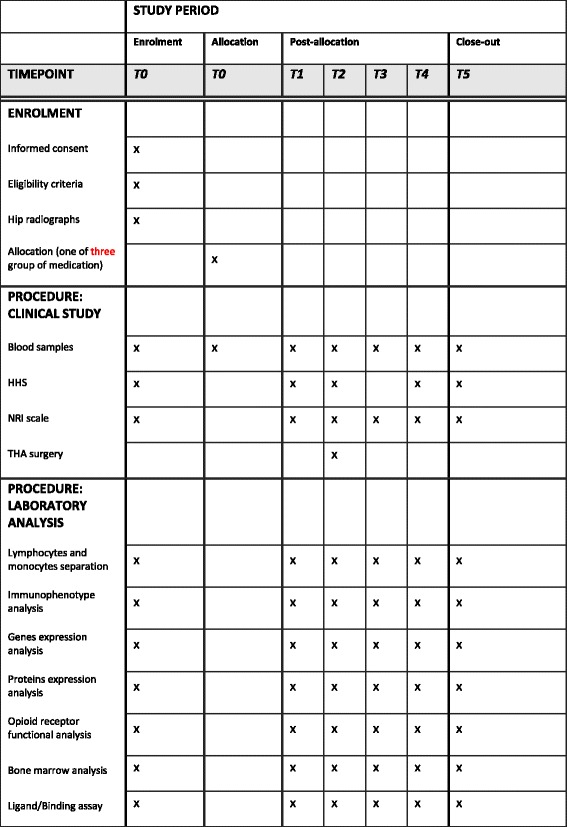
SPIRIT figure

Blood samples will be processed to determine:

- *Lymphocyte and monocyte separation*. Peripheral blood mononuclear cells (PBMCs) will be isolated using a Ficoll density centrifugation gradient. To obtain monocytes, PBMCs will be incubated with anti-CD14 antibody conjugated to magnetic beads (Milteniy Biotech). The CD14 negative fraction will be incubated with magnetic beads conjugated to different lymphocyte populations’ specific antibodies. Purified cells will be utilized for the extraction and subsequent analysis of RNA and proteins.
- *Immunophenotype analysis*. To assess the expression of opiod receptors on white blood cells, peripheral blood will be incubated with fluorochrome-conjugated antibodies, specific for different cell population membrane markers, in combination with anti-opioid receptor antibodies. Red cells will be eliminated by FACS lysing solution (Becton Dickinson) and samples will be acquired and analyzed using a FACScalibur flow cytometer (Becton Dickinson).
- *Gene expression analysis*. RNA will be extracted with TRIZOL reagent (Life Technologies), and cDNA will be synthesized by using SuperScriptIII (Life Technologies). Real Time PCR will be performed following standard protocols, in order to analyze opioid receptor mRNA expression before, during and after treatment.
- *Protein expression analysis*. Protein expression will be analyzed through Western blotting analysis, using opioid-receptor-specific antibodies.
- *Opioid receptor functional analysis*. Cytokines released by lymphocytes in the presence of specific opioid receptor agonists and antagonists will be tested, in order to analyze opioid receptor functionality. Cells will be stimulated and supernatants will be utilized for ELISA analysis.
- *Bone marrow analysis*. Bone marrow biopsy will be fixed for 24 h in buffered 4% formalin and included in paraffin; 3 μm sections will be used for bone marrow morphological and morphometric evaluation through haematoxylin and eosin staining protocol and for cellular immune-phenotypic typization (SPIRIT Fig. 1).

All the biological specimens will be collected and stored following routine laboratory protocol.

#### Study outcomes

The primary outcome is to verify whether or not a presurgical daily opioid dosage for osteoarthritic patients, enrolled for hip replacement, can prevent the chronicization of pain and the insurgence of secondary diseases, in comparison with conventional NSAID or paracetamol treatment. We also want to verify whether different opioid formulations lead to diverse effects and, at the same time, we want to verify whether opioid receptors on the surface of circulating lymphocytes can be considered as biological markers of therapeutic trends.

The secondary outcome measure is the evaluation of functional autonomy, due to the therapy, before and after surgery, calculated using the Harris Hip Score (Additional file 1) and a numeric rating pain scale (Additional file 2).

#### Endpoints

The specific measurement variable corresponds with the analysis of lymphocyte opioid receptor expression and functional characteristics through peripheral blood analysis, in order to consider lymphocyte opioid receptors as diagnostic markers of therapeutic evolution. We will boost experimental data with clinical evaluation, in order to fix the best pre-surgery opioid dosage to prevent pain chronicization. We will compare data from the three groups of medication: 25 mg tapentadol twice daily; 75 mg tapentadol twice daily; NSAIDs or paracetamol in accordance with surgeon’s customary practice. Moreover, we will analyze baseline changes, for individual patients, from time zero to specific time points (T0, day of enrolment and starting therapy; T2, surgery day; T3, the day after surgery; T4, 30 days after surgery; T5, 60 days after surgery).

### Statistics

This is a pilot study and it is not possible to determine sample size because of the lack of necessary quantification parameters. Thus, we will choose 60 patients, 20 for each arm. For descriptive analysis, we will determine central tendency, variability, symmetry and kurtosis. We will show confidence interval, interquartile range and graphic representations for variables and explicates (covariates). Contingency tables with evidence of relative row and column profiles will be designed. Considering inferential statistical analysis, ANOVA and one way, post-hoc (Student’s *t*) comparisons, Bonferroni correction, Scheffè and Tukey methods, least significant difference and honestly significant differences, Chi-Quadro and Fisher tests will be performed. Inferential results with *p* ≤ 0.05 will be considered statistically significant. Any subgroup or adjusted analysis would be planned according to a positive evaluation of the proof-of-concept phase. The statistical analysis of the proof-of-concept phase will be mainly descriptive in nature. The only inferential result will be the 95% confidence interval of sample means and proportions. As a pilot study, we would like to analyze the parameters of this first sample from a clinical point of view. If we judge that the recorded data can assess our ‘proof of concept’, we will ask our statistician to write a statistical analysis plan, in which we will report our clinical and statistical hypothesis and the related sample size calculation, as well as the statistical analysis strategy for primary and secondary endpoint measures, as required by SPIRIT guidelines.

### Data monitoring

Clinical and biological data will be monitored monthly by the sponsor ISAL Foundation. In particular, each month the biologists will share obtained data with the clinicians who will complete the electronic file, filled daily with clinical evaluations. The sponsor will analyze the interim results until the end of the trial. Any spontaneous or unintended adverse event will be reported to the sponsor and managed to limit the patient’s risks. The sponsor will access the final trial dataset to decide the best way to disclose the results.

## Discussion

Chronic pain represents a challenging condition, as it can be disabling, severe and intractable, causing both distress and suffering. Every year, European national health systems spend economic resources on drugs and therapies, often without any clear or permanent result [16–19]. A precise diagnosis is arduous and medical doctors can only rely on patients’ descriptions of symptoms; this is why it is really hard to decide which is the best formulation, for the best antinociceptive efficiency, at different pain stages. Clinicians, before surgery, do not have guidelines to follow on the best biological formulation and dose; they usually have to choose between continuous analgesic treatment or on-demand opiod therapy. In fact, opioids are used in chronic pain therapy but the healing is overtaken by side effects, as well as respiratory and cognitive dysfunctions and immune impairment [20–22]. In particular, opioid therapy is at the centre of a long debate because of its contrasting role in releasing pain and inducing, at the same time, tolerance and addiction [23]. It can be reasoned that the problem lies in the lack of statistical data about the right dose for drug utilization and long-term efficacy. It is not clear whether different opioid formulations can lead to diverse effects, or whether the main goal in this field should be to achieve pain control or better rescue functional abilities. Moreover, the genetic and metabolic processes causing pain conditions are still not confirmed. The absence of objective tests and biological markers, to monitor risk factors and pain development, leads to a deceptive and negative consideration of opioid consumption. However, in this confused background, an excessive inflammatory response seems to have a key role in the pathophysiology of chronic pain, and different opioids or diverse opioids administrations show various effects on immune system, as well as immunosuppression or immunostimulation, or both [24]. In particular, hyperalgesia can be considered the result of synergy across immune, nervous and peptidergic systems. Immune and immune-related cells, such as vascular endothelial cells and keratinocytes, secrete anti-inflammatory cytokine, opioid peptides and proresolution lipid mediators to block pain. Thus, the question is open: is this cooperating mechanism involved in pain defence or in enhancing damage? To answer the question, we must remember that each immune system cell type has a role in the process. For example, mast cells, which release vasodilator mediators, as well as histamine and bradykinin, have been found next to the primary nociceptive neurons and participate in nociceptor sensibilization. However, it is not clear which specific mediators regulate the event [25–27]. Macrophages are normally recruited, in the site of injury, by inflammatory cytokines (i.e. TNF-α, IL-15) and contribute to mechanical allodynia. Thus, macrophages participate in the sensitization of nociceptors and neuropathic pain development, by releasing soluble mediators themselves (i.e. MIP-1α CCR1–CCR5) [28]. Moreover, macrophage depletion partially reduces mechanical and thermal hyperalgesia without alteration of mechanical allodynia [29]. Neutrophil migration to the site of damage is linked to inflammatory pain. These cells are recruited, influenced by afferent neurons during neurogenic inflammation and generate impulses, releasing P substance and calcitonin gene-related peptide. Neutrophil migration is also influenced by IL-1 [30]. The complement system participates in inflammatory hyperalgesia and chronic pain; C5a anaphylotoxin, belonging to the complement cascade, acts as a potent attractant of neutrophils once linked to C5aR1 neutrophil receptors. In rodent models, C5a and C3a injection produces hyperalgesia; C5a and C3a *ex-vivo* application sensitizes C fibres, facilitating neutrophil migration and hyperalgesia and C5a activates the spinal microglia during neuropathic pain [31–34]. Considering lymphocytes, their role in the sensitization of nociceptors is not clear yet. There is evidence that T-helper 1 (Th1) and 2 (Th2) lymphocytes have different functions in the generation of pain: Th1 lymphocytes release pro-inflammatory cytokines (i.e. IFNγ, IL-2) facilitating neuropathic pain, Th2 lymphocytes release anti-inflammatory mediators (i.e. IL-4, IL-10, IL-13) inhibiting the process [35]. Natural killer cells and B lymphocytes are also recruited during inflammation but there is no evidences of their involvement in the development of neuropathic pain, in animal models [36–38]. However, human studies have shown that opioid therapy could functionality influence natural killer cells and B lymphocytes [10, 11], and interferes with pain expression and pathological evolution in osteodegenerative syndrome [39]. Moreover, studies suggest that opioids must be used with care in patients who are already immunosuppressed by disease or by other concurrently administered drugs, because opioid therapy (1.5–4 mg/day) increases μ-opioid receptor (MOR) mRNA levels in lymphocytes of 65% compared with controls and 47% compared with pre-treatment values. Even higher levels (an increase of 142% compared with controls and 135% with pre-treatment values) were observed in patients treated with morphine plus bupivacaine (0.2–0.4 mg/day). Elevation of MOR mRNA levels was confirmed in patients after 24 months of treatment and the percentage of natural killer cells was significantly decreased [15].

At this point, the involvement of immune cells, cytokines, soluble mediators and their specific receptors in the pathophysiology of pain has to be considered as a starting point for a debate. Where does pain pathology take its origin? We could suppose that it is due to an incorrect release of such mediators by the blood cells. It could be possible that the quantity of released factors is not enough for defence. We could also hypothesize an over-release of mediators or an incorrect delivery. It could be possible that the specific receptors, on the target cells, are qualitatively or quantitatively expressed in a nonphysiological way, by producing a persistent sensitivity.

In accordance with these ideas, we propose our protocol to study the elements involved in the process. Specifically, we will focus on the concept that the correlation between pain and the immune system finds its strongest evidence in the presence of opioid receptors on the surfaces of lymphocytes, mast cells and natural killer cells [7, 40–42]. This is a pivotal point, which will lead us to a new analysis of opioid activity, by confuting the past concept of their exclusive action on the central nervous system. The role of exogenous and endogenous opioids in significant reduction of hyperalgesia, induced by peripheral inflammation, is crucial and leads us to propose an innovative diagnostic approach. Specific qualitative or quantitative characteristics of peripheral opioid receptors on the lymphocyte surface, linked with a chronic pain status, will be used as markers of pathology. Their expression and functionality could be relevant for drug selection and tailored dose setting.

To verify our hypothesis, we will choose orthopaedic patients following opioid treatment for hip osteodegenerative pain and selected for hip replacement. This study protocol will help to set up the best therapy based on the lowest efficient dose and economy per patient, during the minimum time period, in order to bypass tolerance and addiction due to opioids. Our study presents an easy and noninvasive diagnostic plan, using peripheral blood samples, in which patients will not be overloaded by clinical tests and will be enlisted during routine clinical visits, by limiting stress, anxiety, costs and social problems.

### Trial status

The trial is currently running; 25 patients have been involved so far and cellular and molecular analysis are ongoing.

## Additional files

Additional file 1: Harris Hip Score protocol. (PDF 84 kb)

Additional file 2: Numeric pain rating scale. (PDF 180 kb)

Additional file 3: SPIRIT check list. (DOCX 52 kb)

## Abbreviations

ELISA: Enzyme-linked immunosorbent assay
MOR: μ-opioid receptor
NSAID: Non-steroidal anti-inflammatory drug
OpMarkArt: Opioids-Markers-Arthroprosthesis
PBMC: Peripheral blood mononuclear cell
PCR: Polymerase chain reaction
Th1: T-helper 1
Th2: T-helper 2
