# Supplementary material for: Lymphocyte opioid receptors as innovative biomarkers of osteoarthritic pain, for the assessment and risk management of opioid tailored therapy, before hip surgery, to prevent chronic pain and opioid tolerance/addiction development: OpMarkArt (Opioids-Markers-Arthroprosthesis) study protocol for a randomized controlled trial
Source: Trials. 2017 Dec 19;18:605. doi: 10.1186/s13063-017-2363-z (PMC5738165; doi:10.1186/s13063-017-2363-z)
Supplement: Supplementary file 1 — Harris Hip Score protocol. (PDF 84 kb) [file 13063_2017_2363_MOESM1_ESM.pdf]

Harris Hip Score

Hip ID:

Study Hip: ☐ Left ☐ Right

Examination Date (MM/DD/YY): / /

Subject Initials: |____|____|____|

Medical Record Number:

Interval: _______________

Harris Hip Score

Pain *(check one)*

☐ None or ignores it (44)

☐ Slight, occasional, no compromise in activities (40)

☐ Mild pain, no effect on average activities, rarely moderate pain with unusual activity; may take aspirin (30)

☐ Moderate Pain, tolerable but makes concession to pain. Some limitation of ordinary activity or work. May require Occasional pain medication stronger than aspirin (20)

☐ Marked pain, serious limitation of activities (10)

☐ Totally disabled, crippled, pain in bed, bedridden (0)

Limp

☐ None (11)

☐ Slight (8)

☐ Moderate (5)

☐ Severe (0)

Support

☐ None (11)

☐ Cane for long walks (7)

☐ Cane most of time (5)

☐ One crutch (3)

☐ Two canes (2)

☐ Two crutches or not able to walk (0)

Distance Walked

☐ Unlimited (11)

☐ Six blocks (8)

☐ Two or three blocks (5)

☐ Indoors only (2)

☐ Bed and chair only (0)

Sitting

☐ Comfortably in ordinary chair for one hour (5)

☐ On a high chair for 30 minutes (3)

☐ Unable to sit comfortably in any chair (0)

Enter public transportation

☐ Yes (1)

☐ No (0)

Stairs

☐ Normally without using a railing (4)

☐ Normally using a railing (2)

☐ In any manner (1)

☐ Unable to do stairs (0)

Put on Shoes and Socks

☐ With ease (4)

☐ With difficulty (2)

☐ Unable (0)

Absence of Deformity (All yes = 4; Less than 4 =0)

Less than 30° fixed flexion contracture	☐ Yes	☐ No
Less than 10° fixed abduction	☐ Yes	☐ No
Less than 10° fixed internal rotation in extension	☐ Yes	☐ No
Limb length discrepancy less than 3.2 cm	☐ Yes	☐ No

Range of Motion (**indicates normal)*

Flexion (*140°) _________

Abduction (*40°) _________

Adduction (*40°) _________

External Rotation (*40°) _________

Internal Rotation (*40°) _________

Range of Motion Scale

211° - 300° (5)	61° - 100 (2)
161° - 210° (4)	31° - 60° (1)
101° - 160° (3)	0° - 30° (0)

Range of Motion Score ____________

Total Harris Hip Score ____________
