# Supplementary material for: Lymphocyte opioid receptors as innovative biomarkers of osteoarthritic pain, for the assessment and risk management of opioid tailored therapy, before hip surgery, to prevent chronic pain and opioid tolerance/addiction development: OpMarkArt (Opioids-Markers-Arthroprosthesis) study protocol for a randomized controlled trial
Source: Trials. 2017 Dec 19;18:605. doi: 10.1186/s13063-017-2363-z (PMC5738165; doi:10.1186/s13063-017-2363-z)
Supplement: Supplementary file 2 — Numeric pain rating scale. (PDF 180 kb) [file 13063_2017_2363_MOESM2_ESM.pdf]

NUMERIC PAIN RATING SCALE

"Please indicate the intensity of current, best, and worst pain levels over the past 24 hours, on a scale of 0 (no pain) to 10 (worst pain imaginable)"

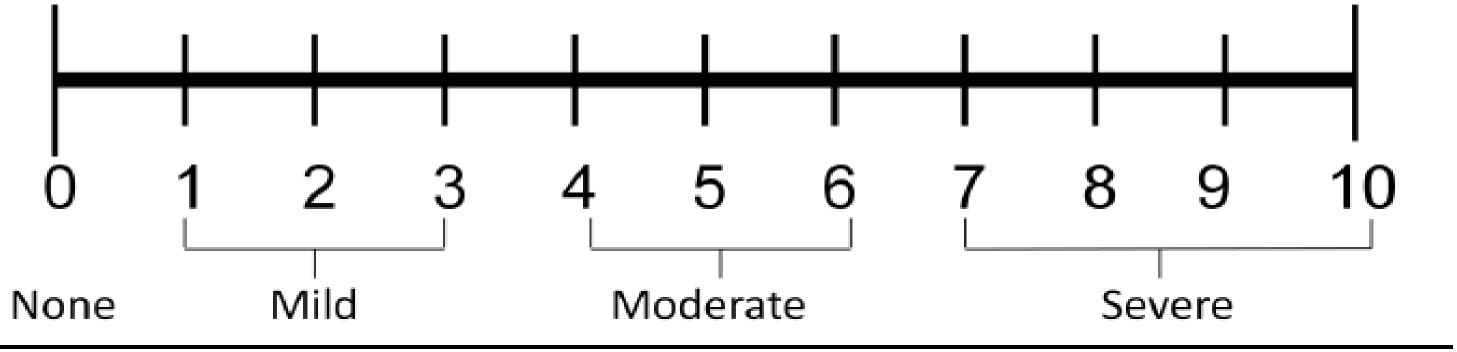

Reference: McCaffery M, Beebe A et all (1989) Pain: Clinical manual for nursing practise.
